# American Indian and Alaska Native veterans in the Indian Health Service: Health status, utilization, and cost

**DOI:** 10.1371/journal.pone.0266378

**Published:** 2022-04-01

**Authors:** Carol E. Kaufman, Laura Grau, Rene Begay, Margaret Reid, Cynthia W. Goss, Bret Hicken, Jay H. Shore, Joan O’Connell

**Affiliations:** 1 U.S. Department of Veterans Affairs (VA), Office of Rural Health (ORH), Veterans Rural Health Resource Center in Salt Lake City, Salt Lake City, UT, United States of America; 2 Centers for American Indian and Alaska Native Health, University of Colorado-Anschutz Medical Campus, Aurora, CO, United States of America; 3 Department of Community and Behavioral Health, Colorado School of Public Health, University of Colorado-Anschutz Medical Campus, Aurora, CO, United States of America; 4 Department of Health Services, Management, and Policy, Colorado School of Public Health, University of Colorado-Anschutz Medical Campus, Aurora, CO, United States of America; 5 Department of Psychiatry, School of Medicine, University of Colorado-Anschutz Medical Campus, Aurora, CO, United States of America; University of Western Australia, AUSTRALIA

## Abstract

**Purpose:**

Many rural American Indian and Alaska Native (AIAN) veterans receive care from the Indian Health Service (IHS). United States Department of Veterans Affairs (VA) has reimbursement agreements with some IHS facilities and tribal programs and seeks to expand community partnerships in tribal areas, but details of how AIAN veterans use IHS are unknown. We aimed to assess the health status, service utilization patterns, and cost of care of veterans who use IHS.

**Methods:**

We used comprehensive and integrated IHS data to compare health status, health service utilization and treatment cost of veterans (n = 12,242) to a matched sample of non-veterans (n = 12,242). We employed logistic, linear, or negative binomial regressions as appropriate, by sex and overall.

**Findings:**

Compared to non-veterans, veterans had lower odds of having hypertension, renal disease, all-cause dementia, and alcohol or drug use disorders, but had similar burden of other conditions. In service utilization, veterans had lower hospital inpatient days; patterns were mixed across outpatient services. Unadjusted treatment costs for veterans and non-veterans were $3,923 and $4,145, respectively; veteran adjusted treatment costs were statistically lower. Differences in significance by sex were found for health conditions and service use.

**Conclusions:**

AIAN veterans, compared to AIAN non-veterans, were not less healthy, nor did they require more intensive or more costly care under IHS. Our results indicate the viability and importance of expanding IHS-VA partnerships in community care.

## Introduction

American Indian and Alaska Natives (AIAN) have served in the United States (U.S.) military since the American Revolution [1], and they have served at disproportionately high levels compared to other race/ethnic groups [2]. This commitment to armed service reflects a long tradition of protecting their land and people [1, 3], even in the context of a historically fraught relationship with the federal government including genocide, broken treaties, aggressive deculturation policies, and forced removal from traditional lands to rural, remote, and small segmented “reservation” areas [4, 5]. The Indian Health Service (IHS) was created in 1955 as a response to federal treaty obligations to provide health to AIAN people [6, 7]. Nonetheless, AIAN health indicators consistently lag–often substantially–behind most other race groups in the U.S., in part a result of the trauma and discriminatory practices carried across generations [4, 8, 9]. AIAN veterans, have fared no better, with studies indicating an excessive burden of both physical and mental health disadvantage [10, 11]. This description of AIAN military service and subsequent veteran disadvantage within the context of historical colonialism and prejudice has parallels in other settler societies, including Canada, Australia, and New Zealand [12]. Indigenous people have served at high levels–a metric of inclusion into society, but have also faced limited resources to support health and well-being at re-integration [13, 14]. Indeed, Sheffield has asserted that the experiences of returning Indigenous soldiers demonstrated both the advances in access to benefits in view of military service, but also the very real limitations imposed on these soldiers to access rewards structured to exclude them [12]. The AIAN veteran case provides an example of this tension.

Many rural AIAN veterans can access care through both the U.S. Department of Veterans Affairs (VA) and IHS. Kramer and colleagues found that 25% of AIAN veterans used both VA and IHS care, while about 46% used IHS services only [15]. In 2010, IHS and VA signed an updated memorandum of understanding that encourages coordination, collaboration, and resource sharing between the agencies [16]. Subsequent provisions allow IHS facilities and Tribal Health Programs to enter into an agreement for the reimbursement of veteran direct care services (“reimbursement agreement”). However, each agreement must be negotiated individually by tribe as no universal reimbursement agreement exists [17]. In total, in 2020, VA had 74 IHS sites under VA-IHS reimbursement agreements and 116 individually signed VA-Tribal Health Program reimbursement agreements across 574 federally recognized tribes (personal communication, M. Slusser, 2020 Jun 23). In the past decade, VA financial support for AIAN veteran care has continued to grow in incremental ways [18]. At the same time, VA priorities include the expansion of partnerships—collaborative working relationships—with viable community health services for some veteran care [19]. Together, these efforts may quickly foreground policy and practice of IHS care for AIAN veterans.

Despite the progress made in increasing collaboration between the two systems, research regarding optimization of care has been slim. Since health records are not shared between the agencies, utilization patterns of dual users have not been explored, with two notable exceptions. Using data from 2002–2003, Kramer and associates linked IHS and VA records and found that the likelihood of dual use increased with the number of diagnoses, and compared to those using IHS only, dual users were more likely to use intensive specialty health care services, suggesting medical need contributes to dual use [15, 20]. In addition to type of care required, differential patterns of use across the two systems has also been shown to likely reflect distance or transportation barriers, and perceptions of cultural competency or discrimination in care [20, 21]. In another study, Kramer and associates linked IHS, VA, and Medicare/Medicaid records from 2009–2012 for a sample of rural veterans (n = 88) to examine home-based primary care [22]. They found that home-based primary care reduced hospitalizations and emergency room visits for IHS and non-IHS beneficiaries in the rural sample, supporting the promise of increased VA-IHS partnership.

While efforts to link data comprehensively across the two systems have not materialized, the need for coordinated care has likely increased. Research using data from 2008 indicated that rural AIAN veterans accessing VA care were more likely to have experienced combat, have higher ratings of service-connected disability, and higher mean number of visits compared to their rural non-Native counterparts [23]. Other work, based on a national sample from years 2011 and 2012, found that compared to rural non-Hispanic White veterans, rural AIAN veterans reported significantly worse health across multiple domains [11]. The emerging picture indicates a critical care gap for AIAN veterans, even within an evolving policy context. Improving care for AIAN veterans across VA and IHS systems, however, requires consideration of their differing service ecologies.

Differences between IHS and VA in operational systems and in mandate shape veteran health care experiences and associated costs. IHS is charged with providing health care to 2.6 million AIANs [24]. As a result of numerous treaty agreements, based in part on the exchange for tribal lands and natural resources, the U.S. government has a trust responsibility to provide health services for AIANs. To meet this responsibility, the U.S. government established the IHS health care system to provide health services at no cost to members of federally recognized tribes [6]. Tribal members are eligible to use IHS funded services (e.g., inpatient, outpatient, and home services) throughout their lifetime. IHS funds support the provision of services by IHS and tribes in 45 hospitals, 335 health centers, 134 Alaska Native village clinics, and 83 health stations. IHS and tribal providers supplement IHS funding with reimbursement for provided services from Medicare, Medicaid, VA, and private insurers; funding from Tribes; and grants [6]. While some hospitals and clinics are located in urban areas, most are located on or near tribal reservations.

The IHS is not an insurance program, but instead receives appropriations from Congress. It is chronically under-funded [25]; IHS expenditures were $4,078 per capita in fiscal year (FY) 2017 [26], and although this estimate does not include all spending associated with patient care, it is about half of what is spent on federal prisoners and substantially lower than per capita spending for the U.S. general population ($10,742 in 2017) [27, 28]. As a result, the IHS is forced to ration care in a number of ways in an attempt to maintain at least basic services throughout the year [29]. IHS resources are further strained by provider shortages and by community-level factors that compromise service use and health (e.g., low household income, rural geography) [30–38]. AIANs experience significant morbidity and mortality due to chronic disease. AIAN all-cause mortality rate was found to be 46% higher than that of non-Hispanic Whites living in geographic locations served by IHS, due in part to higher rates of premature death caused by heart disease, stroke, and diabetes among AIANs [39–42].

The VA, in contrast, has a budget of $68 billion for health care to serve 9 million veterans, with a per capita expenditure of approximately 3 times that of IHS [43, 44]. This relatively large amount in part is due to the chronic and complex care needed for veterans as they age, leveraged by general political support for veterans. VA has 1,243 facilities across the country, mostly located in urban areas, though the more than 1,000 outpatient facilities, including Community-Based Outpatient Clinics, continue to expand to rural locations [44]. Not all veterans are eligible for VA services; eligibility is based on a number of criteria, which could include duration of service, discharge status, service-connected disability status, priority group membership (e.g., prisoner-of-war), and income. In total, about 48% of all veterans are enrolled in VA health care [45]. VA has recently accelerated its efforts to expand access to veterans, especially those in rural areas. One of the central mechanisms for this expansion is the Veterans Community Care Program, an outgrowth of VA Maintaining Internal Systems and Strengthening Integrated Outside Networks (MISSION) Act of 2018. Expansion is likely to encompass identification and engagement of local health care partners who can meet VA standards of care, which may include IHS Service Units operating on or near tribal reservations and Alaska Native villages.

Here, we analyze data from IHS to assess the health status, service utilization patterns, and cost of care of AIAN veterans in the IHS system. Absent system-wide VA-IHS linked records, our goal is to compare the health status, utilization, and cost of care of AIAN veterans to their AIAN non-veteran counterparts in order to inform the process of expanding VA support for local IHS services in the care of rural AIAN veterans. Because health conditions and care utilization patterns differ substantially by sex among veterans, our analyses disaggregate results by sex also [46–49]. Our data provide a snapshot of IHS health care for veterans, revealing the initial insights into characteristics, use, and cost of services for this population. We hypothesize that veterans enrolled in this system will be less healthy, use care more frequently, and cost more than non-veterans accessing IHS care because of compromised health related to military service and fragmented health care. These results will be among the first to describe AIAN veteran health experiences within the IHS system.

## Methods

### Data

Data were extracted from IHS Improving Health Care Delivery Data Project infrastructure. (See S1 File and O’Connell et al. 2014 [31] for details.) Since 2010, IHS Data Project team has collaborated with IHS to create a longitudinal data infrastructure that houses health status, service utilization, and treatment cost data for over 640,000 AIANs who reside throughout the U.S., representing nearly 30% of AIANs who use IHS services. Through use of these existing data, we were able to study the health experience of a large number of AIAN veterans. For this analysis, we analyzed FY 2013 data; they are the latest available data from this project. A request for updated data is in process.

The data infrastructure includes information for a sample of AIANs who lived in 15 IHS Service Units, which are geographic areas designated by IHS (hereafter referred to as project sites). The study sample represents the broad geographic distribution of the AIAN population. Using regional classifications employed elsewhere, one project site was located in the East, four in the Northern Plains, two in the Southern Plains, five in the Southwest, two in the Pacific Coast, and one in Alaska. The IHS Data Project population was identified by project site, rather than by random sampling, to create important site treatment cost measures not available elsewhere (e.g., service cost estimates described below). The IHS service delivery system includes IHS operated hospitals and clinics, tribally operated hospital and clinics, and urban Indian clinics. The 15 project sites include 9 sites where IHS directly provides health services and 6 sites that are tribally operated. The IHS Data Project includes little data for urban Indian clinics; we refer to IHS and tribal (I/T) services and other (non-I/T) services below. The project population was comparable to the national IHS service population in age and sex [50].

IHS electronic data from three sources are included in the data infrastructure. The sources, described below, include the: 1) National Data Warehouse for patient registration and IHS and Tribal (I/T) service utilization data; 2) Purchased/Referred Care data for patient service utilization data at non-I/T providers, paid for by IHS and Tribal Health Programs; and 3) Centers for Medicare & Medicaid Services Cost Reports (Cost Reports) for project site cost data. Patient data from National Data Warehouse and Purchased/Referred Care sources are linked though use of computer-generated numbers that are unique to each patient. Hereafter, we use the general term “patient data” to refer to these two sources of data.

### Project approvals

Project personnel collaborate with IHS and the Tribal organizations that participate in IHS Data Project. This collaboration takes place through the project’s Collaborative Network, which includes three advisory committees (i.e., Steering, Project Site, and Patient) and a process to obtain written approvals from IHS National Institutional Review Board (IRB), Tribal IRBs, Tribal Councils, or Tribal Authorities, in addition to the collaborating university’s IRB, the Colorado Multiple Institutional Review Board. Specific approving Tribal entities are not named to protect community confidentiality, as requested by participating sites.

### Study population

We identified 12,428 AIAN adults who obtained services at least once during FY 2011–2013 and who also self-identified as veterans. We created a matched sample of non-veterans who were matched with veterans by birth year, sex, and project site. Since age and sex influence health status, service utilization, and treatment costs, and since the age and sex distribution of AIAN veterans did not match that of AIAN non-veterans in the FY2013 Data Project population, matching samples was important to ensure meaningful comparisons. We identified a non-veteran match for 98.5% (n = 12,242) of the veterans. Over 60% of the veterans who were not matched with a non-veteran in their project site were aged 85 years and older. Analyses conducted for this report include FY 2013 data for the 12,242 veterans and the 12,242 matched non-veterans. Using the 2013 National Center for Health Statistics urban-rural classification scheme, approximately 60% of the sample resided in small, micropolitan, or non-core counties [51].

### Measures

#### Demographic and health status

Patient data provided information on age, sex, project site, health coverage, and veteran status. Data Project algorithms, developed from national references, were used to identify adults with diabetes, end-stage renal disease, and all-cause dementia using data on ICD-9-CM diagnoses, procedure codes, medication use, and blood glucose control included in the inpatient and outpatient service utilization records [52–54]. Sightlines^TM^ DxCG Risk Solutions software [55] was used with patient data on ICD-9-CM diagnoses to identify patients diagnosed with other conditions, including hypertension, cardiovascular disease (CVD), chronic obstructive pulmonary disease, asthma, malignant cancers, liver disease, and mental health and substance use disorders.

#### Health service utilization

Annual service utilization measures were created from patient data. Sources of inpatient data were combined to create two hospital inpatient utilization measures that included utilization at I/T and non-I/T hospitals: the percentage of adults with one or more hospitalizations and the number of inpatient hospital days. Patient data on clinic and provider type were used to report utilization for nine types of I/T outpatient services based on point of care (e.g., emergency room, dental, etc.); patient data were also used to report on outpatient visits at non-I/T providers. Data on I/T dispensed medications were used to calculate the average number of prescriptions filled per adult.

#### Treatment costs

IHS treatment costs were estimated from patient I/T utilization data, project site cost data for I/T services, and payment data for non-I/T services obtained by patients. The Cost Report data are prepared by IHS financial consultants and collected to create two sets of Medicare and Medicaid reimbursement encounter rates for I/T provided services. These data include site-specific costs for personnel salaries and benefits, facilities, equipment, operational costs (e.g., heating, electricity), medical and other supplies, and medications, according to U.S. Office of Management and Budget regulations. We estimated site-specific costs for a broad array of I/T-provided services using Cost Report and patient I/T utilization data that were supplemented by data obtained from the project sites.

Treatment costs for I/T-provided services for each person were estimated based on their utilization of I/T services (e.g., number of emergency, primary care, and behavioral health visits) during FY 2013 and the estimated average costs of providing those services in the site where they lived during the same year. IHS and tribes’ paid amounts for Purchased/ Referred non-I/T services were used to estimate costs for those services. Hereafter, we refer to FY 2013 annual treatment costs for each person as the sum of their estimated costs for I/T and non-I/T services during that year. It is important to note that our treatment cost measures do not include costs for services obtained at non-I/T providers that are not paid for through Purchased/Referred Care (e.g., VA services, dialysis services). Tribal members in IHS care do not pay for I/T provided or Purchased/Referred covered services.

#### Analysis

We used SAS v.9.4 software [56] for variable construction and statistical analyses. The non-veteran matched sample was created using the SAS GMATCH macro [57]. Based on national VA statistics, we anticipated the age distribution of AIAN veterans to vary by sex and provide results stratified by sex [2]. Statistically significant differences between veterans and non-veterans were determined using logistic regression for dichotomous health status and utilization measures; negative binomial regression for service utilization measures; and generalized linear regression with a gamma distribution and a log-link function for cost measures, adjusting for age, sex, and project site.

## Results

Table 1 provides the age and sex of the sample by veteran status. Females represented 10.2% (n = 1,248) of the veterans and tended to be younger than the male veterans; 52.9% of female veterans were under 45 years old compared to 19.3% among the male veterans. The percentage of veterans aged 65 years and older was 10.4% among females and 41.3% among males.

**Table 1:** American Indian and Alaska Native veterans and non-veterans by age and sex. Fiscal year 2013.

	Female				Male				All adults			
	Veterans		Non-Veterans		Veterans		Non-Veterans		Veterans		Non-Veterans	
Age group	Number	Percent (column)	Number	Percent (column)	Number	Percent (column)	Number	Percent (column)	Number	Percent (column)	Number	Percent (column)
18–34	391	31.3%	390	31.3%	937	8.5%	937	8.5%	1,328	10.9%	1,327	10.8%
35–44	269	21.6%	276	22.1%	1,183	10.8%	1,195	10.9%	1,452	11.9%	1,471	12.0%
45–54	295	23.6%	289	23.2%	1,787	16.3%	1,779	16.2%	2,082	17.0%	2,068	16.9%
55–64	163	13.1%	164	13.1%	2,549	23.2%	2,542	23.1%	2,712	22.2%	2,706	22.1%
65+	130	10.4%	129	10.3%	4,538	41.3%	4,541	41.3%	4,668	38.1%	4,670	38.2%
All ages	1,248	100.0%	1,248	100.0%	10,994	100.0%	10,994	100.0%	12,242	100.0%	12,242	100.0%

The morbidity burden of veterans and non-veterans is described in Table 2. Among veterans, the prevalence of hypertension, diabetes, and CVD was 47.6%, 31.9%, and 24.4%, respectively. Nearly 9% had renal disease and approximately 8% had chronic obstructive pulmonary disease. The prevalence of all-cause dementia was 2.7% and 29.1% had one or more mental health or substance use disorders. Overall, nearly 18% were diagnosed with one or more mental health disorders; 8.2%, one or more alcohol or drug use disorders; and 11.5%, tobacco use disorders.

**Table 2:** Health status of American Indian/Alaska Native veterans and non-veterans by sex. Fiscal year 2013.

Health condition	Females			Males			All adults		
	Prevalence^1^		Adjusted OR^2^ (95% CI)	Prevalence^1^		Adjusted OR^2^ (95% CI)	Prevalence^1^		Adjusted OR^2^ (95% CI)
	Veterans	Non-Veterans		Veterans	Non-Veterans		Veterans	Non-Veterans	
**Physical conditions**									
Hypertension	21.6%	31.1%	0.51 (0.41, 0.63)	50.6%	51.8%	0.94 (0.89, 1.00) *^5^	47.6%	49.7%	0.90 (0.86, 0.95) ***
Diabetes	16.0%	21.2%	0.67 (0.54, 0.83) ***	33.7%	34.1%	0.98 (0.93, 1.04)	31.9%	32.8%	0.96 (0.90, 1.01)
Cardiovascular disease	10.7%	11.9%	0.88 (0.67, 1.13)	25.9%	25.9%	1.09 (0.94, 1.07)	24.4%	24.5%	0.99 (0.93, 1.06)
Renal disease	2.3%	3.9%	0.58 (0.36, 0.93) *	9.3%	10.3%	0.89 (0.81, 0.98) *	8.6%	9.6%	0.88 (0.80, 0.96) **
Chronic obstructive pulmonary disease	3.0%	3.7%	0.79 (0.50, 1.23)	8.5%	8.6%	0.99 (0.90, 1.10)	7.9%	8.1%	0.98 (0.89, 1.08)
Asthma	9.4%	10.0%	0.93 (0.71, 1.21)	5.1%	5.5%	0.92 (0.81, 1.03)	5.5%	6.0%	0.92 (0.82, 1.02)
Malignant cancer^4^	1.0%	2.5%	0.41 (0.21, 0.78) **	4.8%	4.6%	1.05 (0.92, 1.19)	4.4%	4.4%	1.01 (0.89, 1.14)
Liver disease	3.8%	5.0%	0.74 (0.50, 1.10)	4.3%	4.2%	1.03 (0.91, 1.18)	4.2%	4.2%	1.00 (0.88, 1.13)
End-stage renal disease	-	-		1.3%	2.0%	0.62 (0.50, 0.77) ***	1.2%	1.8%	0.64 (0.52, 0.79) ***
**Psychiatric conditions**									
All-cause dementia	1.2%	1.6%	0.83 (0.36, 1.93)^3^	2.8%	3.4%	0.84 (0.71, 0.99) *	2.7%	3.2%	0.84 (0.71, 0.99) *
Mental health or substance use disorder	31.8%	34.5%	0.88 (0.74, 1.04)	28.8%	28.8%	0.999 (0.94, 1.06)	29.1%	29.4%	0.99 (0.93, 1.04)
Mental health disorder	26.1%	27.3%	0.94 (0.78, 1.12)	16.9%	16.2%	1.05 (0.98, 1.13)	17.8%	17.3%	1.04 (0.97, 1.11)
Depression	17.0%	18.0%	0.93 (0.76, 1.15)	9.3%	9.2%	1.02 (0.93, 1.12)	10.1%	10.1%	1.00 (0.92, 1.09)
Depression and other mental health condition	9.1%	8.8%	1.03 (0.78, 1.36)	4.2%	3.7%	1.13 (0.99, 1.30)	4.7%	4.3%	1.11 (0.98, 1.26)
Other mental health disorders without depression	9.1%	9.4%	0.97 (0.74, 1.28)	7.5%	7.0%	1.08 (0.98, 1.20)	7.7%	7.3%	1.07 (0.97, 1.18)
Alcohol or drug use disorder	6.5%	7.6%	0.84 (0.62, 1.14)	8.4%	9.6%	0.85 (0.77, 0.94) ***	8.2%	9.4%	0.85 (0.78, 0.93) ***
Alcohol use disorder	4.5%	5.3%	0.84 (0.58, 1.21)	7.3%	8.3%	0.88 (0.79, 0.97) *	7.1%	8.0%	0.87 (0.79, 0.96) **
Drug use disorder	2.8%	4.3%	0.65 (0.42, 1.00)	2.0%	2.5%	0.78 (0.65, 0.94) **	2.0%	2.7%	0.76 (0.64, 0.90) **
Tobacco use disorder	9.1%	11.5%	0.77 (0.59, 1.00) *^5^	11.7%	10.6%	1.12 (1.03, 1.22) *	11.5%	10.7%	1.08 (0.99, 1.17)

Veterans, compared to non-veterans, had lower odds of having hypertension [adjusted odds ratio (AOR) 0.90; 95% CI:0.86–0.95], renal disease (AOR 0.88; 95% CI:0.80–0.96), end-stage renal disease (AOR 0.64; 95% CI:0.52–0.79), all-cause dementia (AOR 0.84; 95% CI:0.71–0.99), and alcohol or drug use disorders (AOR 0.85; 95% CI:0.78–0.93). They had similar odds of having of diabetes, CVD, chronic obstructive pulmonary disease, asthma, malignant cancer, liver disease, depression, and other mental health disorders.

Differences in health status between veterans and non-veterans varied by sex. Among females, veterans had lower odds of having diabetes (AOR 0.67; 95% CI: 0.54, 0.83), renal disease (AOR 0.58; 95% CI: 0.36, 0.93), malignant cancer (AOR 0.41; 95%CI: 0.21, 0.78), and tobacco use disorders (AOR 0.77; 95%CI: 0.59, 0.996) than non-veterans. Among males, veterans had lower odds of hypertension (AOR 0.94; 95% CI: 0.89, 1.00), renal disease (AOR 0.89; 95% CI: 0.81, 0.98), end stage renal disease (AOR 0.62; 95% CI: 0.50, 0.77), all cause dementia (AOR 0.84; 95% CI:0.71, 0.99), alcohol or drug use disorders (AOR 0.85; 95% CI: 0.77, 0.94), and tobacco use disorder (AOR 1.12; 95% CI: 1.03, 1.22).

Utilization patterns, shown in Table 3, were generally similar by veteran status. During FY 2013, 6.6% of veterans, compared to 7.1% of non-veterans, had one or more hospitalizations at I/T hospitals and non-I/T hospitals. Veterans did not have statistically lower adjusted odds of having one or more hospitalizations than non-veterans. The average number of days veterans spent in the hospital, associated with these admissions, was 0.52, approximately one-half day per adult, lower than non-veterans (0.58). The adjusted incident rate ratio (AIRR) was 0.82 (95% CI: 0.68, 0.98). While over half of the veteran’s admissions (53.8%,) were at non-I/T hospitals, the majority of hospital days were at I/T hospitals (56.2%) (S1 Table in S1 File).

**Table 3:** Hospital inpatient and outpatient service utilization among American Indian/Alaska Native veterans and non-veterans, by sex, at IHS and Tribal (I/T) health facilities and non-I/T facilities. Fiscal year 2013.

	Female			Male			All adults		
	Veterans	Non-Veterans		Veterans	Non-Veterans		Veterans	Non-Veterans	
**Hospital inpatient utilization at I/T and non-I/T hospitals** ^1^									
	**Percent**	**Percent**	**Adjusted OR**^2^ **(95% CI)**	**Percent**	**Percent**	**Adjusted OR**^2^ **(95% CI)**	**Percent**	**Percent**	**Adjusted OR**^2^ **(95% CI)**
One or more admissions	5.2	6.4	0.80 (0.56, 1.15)	6.7	7.2	0.93 (0.82, 1.04)	6.6	7.1	0.91 (0.82, 1.02)
	**Average number**	**Average number**	**Adjusted IRR**^3^ **(95% CI)**	**Average number**	**Average number**	**Adjusted IRR**^3^ **(95% CI)**	**Average number**	**Average number**	**Adjusted IRR**^3^ **(95% CI)**
Inpatient days	0.26	0.34	0.67 (0.37, 1.21)	0.55	0.61	0.82 (0.68, 0.99)*	0.52	0.58	0.82 (0.68, 0.98)*
**Outpatient services**									
	**Average number**	**Average number**	**Adjusted IRR**^3^ **(95% CI)**	**Average number**	**Average number**	**Adjusted IRR**^3^ **(95% CI)**	**Average number**	**Average number**	**Adjusted IRR**^3^ **(95% CI)**
**I/T outpatient services**									
Emergency department ^1^	0.65	0.64	1.06 (0.90, 1.26)	0.53	0.54	1.00 (0.94, 1.07)	0.54	0.55	1.01 (0.95, 1.07)
Urgent ^1^	0.51	0.41	1.32 (1.10, 1.58) **	0.33	0.32	1.07 (0.99, 1.15)	0.35	0.33	1.09 (1.02, 1.17)*
Primary care/general	3.02	3.19	0.96 (0.86, 1.06)	2.81	2.82	0.99 (0.95, 1.02)	2.83	2.86	0.98 (0.95, 1.02)
Specialty	0.34	0.38	0.90 (0.68, 1.20)	0.45	0.45	1.01 (0.93, 1.10)	0.44	0.44	1.00 (0.92, 1.09)
Dental	0.66	0.62	1.04 (0.86, 1.27)	0.64	0.53	1.20 (1.11, 1.28)***	0.64	0.54	1.18 (1.10, 1.26)***
Education, case management, and advanced practice pharmacy	0.35	0.30	0.97 (0.72, 1.31)	0.58	0.55	1.03 (0.94, 1.13)	0.56	0.52	1.02 (0.93, 1.11)
Behavioral health	0.35	0.25	1.52 (0.97, 2.39)	0.18	0.18	1.07 (0.87, 1.32)	0.20	0.19	1.10 (0.91, 1.33)
Physical therapy	0.19	0.14	1.49 (0.85, 2.63)	0.25	0.22	1.17 (0.94, 1.46)	0.25	0.21	1.21 (0.98, 1.48)
Home	0.03	0.13	0.26 (0.10, 0.65) **	0.10	0.16	0.68 (0.51, 0.91)**	0.10	0.16	0.61 (0.47, 0.81)***
Dispensed medications	17.94	21.62	0.91 (0.79, 1.05)	26.04	27.73	0.95 (0.90, 1.00)	25.21	27.10	0.95 (0.91, 1.00)*^4^
**Non-I/T outpatient services** ^1^									
Outpatient visits	0.16	0.16	0.67 (0.40, 1.13)	0.31	0.29	1.13 (0.96, 1.33)	0.30	0.28	1.09 (0.93, 1.27)

* p<0.05

** p<0.01, and

*** p<0.001.

OR: Odds Ratio; IRR: Incident Rate Ratio; CI: Confidence interval.

^1^ Two sites did not provide hospital inpatient or emergency department services and one site did not provide urgent care services; adults from those sites were excluded from analyses of the related utilization measures. One site had incomplete Purchased/Referred Care data and adults from that site were excluded from analyses of hospital inpatient utilization and non-I/T outpatient services.

^2^ Statistical differences in the percent with one or more hospitalizations were assessed using logistic regression, adjusted for age, sex, and site. Due to small sample sizes, some regressions included only age and/or sex adjustments.

^3^ Statistical differences in other service utilization measures were assessed using negative binomial regressions, adjusted for age, sex, and site. Due to small sample sizes, some regressions included only age and/or sex adjustments.

^4^ Confidence interval includes 1.00 due to rounding in the hundredths place.

Patterns of use of particular services were also similar by veteran status, including average number of visits to emergency departments, primary care, and behavioral health (Table 3). Compared to non-veterans, veterans had higher rates of use of urgent care and dental services with AIRRs of 1.09 (95% CI: 1.02–1.17) and 1.18 (95% CI: 1.10–1.26), respectively. Veterans had statistically lower rates of use of home services (AIRR 0.61; 95% CI: 0.47–0.81). The average number of medications dispensed for veterans by I/T pharmacies was 25.21. Veterans had lower rates of use of medications than non-veterans (AIRR 0.95; 95% CI: 0.91–1.00). Veterans had on average 0.30 outpatient visits at non-I/T providers, similar to non-veterans. By sex, female veterans, compared to female non-veterans, had a statistically higher rate of use of urgent care (AIRR 1.32; 95% CI: 1.10–1.58) and a lower rate of use of home services (AIRR 0.26; 95% CI: 0.10–0.65). Outpatient dental and home service utilization differences between male veterans and non-veterans were similar to those of all veterans and non-veterans.

**Fig 1:**
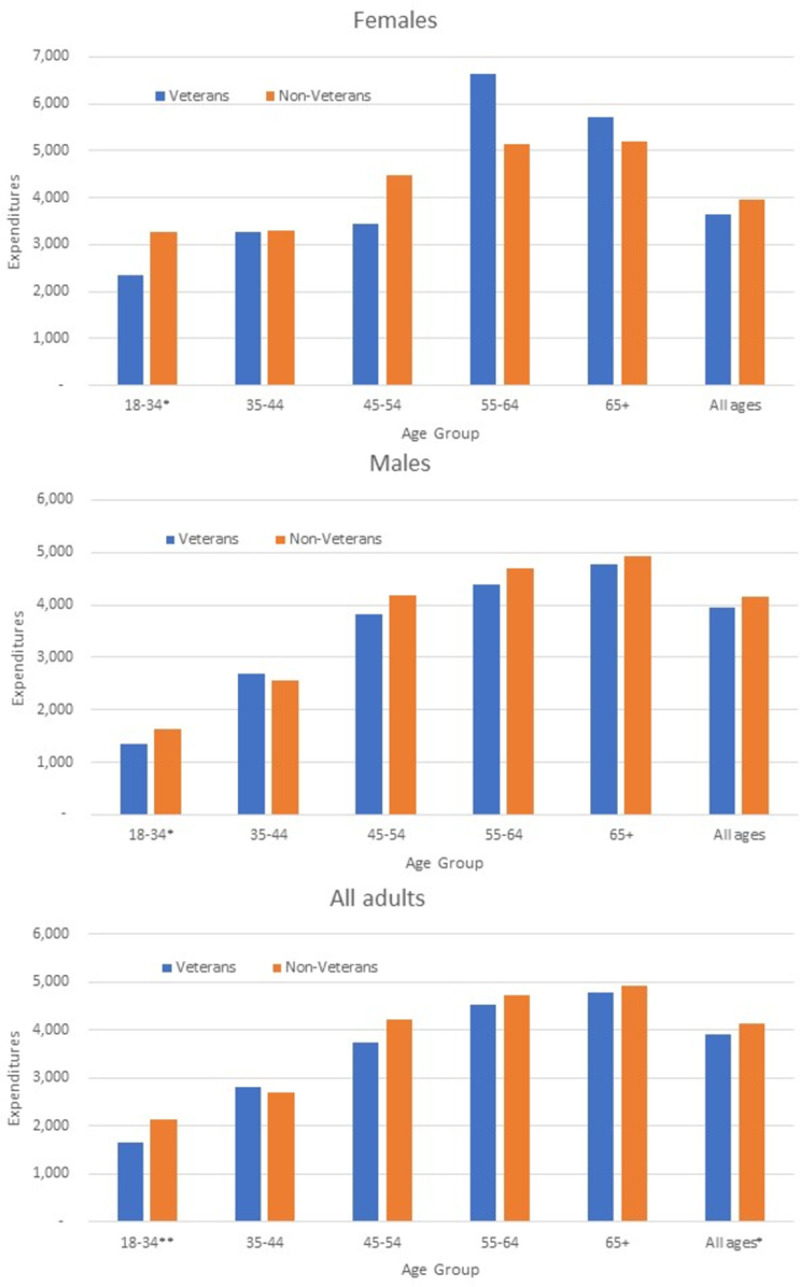
Average annual treatment cost estimates for American Indian/Alaska Native veterans and non-veterans by gender and age group. Fiscal year 2013.

We found few differences in cost of care between veterans and non-veterans using IHS. During FY 2013, unadjusted treatment costs for veterans were $3,923 and for non-veterans were $4,145 (Fig 1), a difference of approximately $200. These costs tended to increase with age. The unadjusted treatment costs for veterans younger than 35 years old were $1,660, while costs for those aged 65 years and older were over $4,500.

Veteran adjusted treatment costs for all ages were significantly lower (p<0.05) than non-veteran costs. Among both females and males of all ages, adjusted treatment costs for veterans were similar to those for non-veterans. By age group, the only statistically significant difference found overall was among those aged 18–34 years, with veterans having had lower adjusted treatment costs compared to non-veterans (p<0.01). However, among those aged 18–34 years, adjusted costs for veterans were significantly lower than costs for non-veterans for both females (p<0.05) and males (p<0.05). About 40% of AIAN veterans did not have Medicare, Medicaid, or private insurance (no information was available about VA coverage). The likelihood of having Medicare, Medicaid, or private insurance was similar for veterans and non-veterans (S1 Table in S1 File).

## Discussion

AIAN peoples serve in the military at rates higher than any other race group except Pacific Islanders [58], and often in positions considered the most dangerous [59]. Like other veterans, they deserve the best health care for serving their country. This analysis is among the first to consider the health status, service use patterns, and cost of care for AIAN veterans accessing services through the IHS delivery system, where so many AIAN veterans receive health care.

Our hypotheses were not supported; our results indicate that veterans did not appear to be more ill, use services more, or cost more for the care they received compared to non-veterans using IHS services. Indeed, in some cases, they appeared to be healthier and accessed IHS care less frequently. While treatment costs for veterans were found to be statistically lower than costs for non-veterans, the difference was modest, approximately $200. These findings contrast with other national studies on veteran health, which have found veterans to be less healthy than the civilian population [60, 61]. At least two explanations are possible. First, AIANs in IHS service areas tend to be significantly less healthy than the rest of the U.S. population [36, 42, 62, 63]. Our results could reflect the poor health status of the AIAN population generally [25, 64], the slightly better health status of AIAN veterans compared to AIAN non-veterans, and, by extension, the inadequacy of IHS resources to ameliorate disparities in health. Second, patients with complex health needs might be more likely to use VA health services or non-I/T specialty care, not paid for through the Purchased/Referred Care program, thus deflating the overall intensity of services and cost associated with AIAN veteran care within the IHS delivery system. It is possible that both explanations are at play; our results speak to the unique—and precarious—health care context of AIAN peoples, both veteran and civilian, not easily captured in single-system analyses.

Our results further propel arguments for expanding reimbursement agreements between tribes and IHS with VA and enhancement of the coordination of care between these two systems. First, VA’s mission is to care for qualified veterans who have served their country. IHS represents an important service partner often more proximal and embedded in the community of rural AIAN veterans than VA. Without reimbursement agreements, IHS is taking on the care of veterans without the full complement of resources. Second, our results show that AIAN veterans, compared to AIAN non-veterans, were not more costly and do not, overall, obtain care that is more intensive. Finally, increasing the equity of reimbursement policies across IHS facilities could improve care for AIANs generally by ameliorating IHS financial burdens associated with veteran care and increasing available resources for overall medical infrastructure. While the contribution of other payers such as Medicare or Medicaid should be considered in the mix of financial viability, VA financial support may increase quality of care for veterans and non-veterans alike who use IHS services. Continuing to find ways to enhance care coordination between these systems becomes even more critical with expansion of this partnership since AIAN veterans will likely continue to receive care both from VA and IHS; care coordination will be key to complementary treatment of dual utilizers.

Our results also have implications for community partnerships in providing care for veterans not easily served by urban-centered VA operations. To the extent that care at IHS facilities meets VA quality of care requirements for basic or routine services (e.g., diabetes management, cancer screening, CVD assessment), more IHS facilities may serve as useful partners to provide such services to veterans residing in remote rural areas where distance or transportation are imposing barriers to VA care. Our findings can inform the scope and types of care VA could support for optimal impact.

Finally, our findings contribute to the literature examining the tensions of race, historical trauma, and veteran benefits for Indigenous veterans cross-nationally [12, 65]. The IHS-VA agreements have been a platform to facilitate resources for veteran care at IHS facilities. Our work suggests the importance of advancing that partnership, and thus advancing equity in care to AIAN veterans even across systems designed, sometimes explicitly but also indirectly, to entrench inequity and historical injustices [6]. In short, our results describe a pathway and inform a model for policy for other countries as they work towards achieving health equity for Indigenous veterans.

Our findings should be viewed with some caution. These IHS data did not indicate which veterans were also eligible for or used VA care or other care outside of IHS. They also did not include those who did not use IHS at all during the study period. Thus, we cannot ascertain relative health status across users and non-users, so our analysis does not present a full picture of care for some. While sites were accounted for in analysis, they vary in number of veterans served. The health status, service utilization, and treatment cost results are based on cross-sectional data limited to one year; the interpretation of findings must remain descriptive, not causal. Our results also do not account for current reimbursement agreements. Indeed, these data are from 2013; however, they are unique in their detail and comprehensiveness, and until the recent pandemic, our findings likely represent patterns sustained since FY 2013. Our data are complex linked administrative data associated with care and do not contain the details that may be obtained from a medical record review. The lack of complete medical information on the study sample may have biased the prevalence of conditions downward (i.e., some health conditions for a patient may not have been recorded in the administrative data). Additionally, these data do not include information on health care services and related costs that were obtained at non-I/T facilities and not paid for through the Purchased/Referred Care program (e.g., VA services, dialysis services). Sites also varied by types of services provided, proportion of veterans served, distance to non-I/T facilities, and completeness of data.

Despite these limitations, these analyses provide important insights to a population largely ignored in the literature. The unique and comprehensive dataset detailed health status, utilization, and cost for a large and geographically diverse sample of AIAN veterans using IHS, finding more similarities than differences between AIAN veterans and non-veterans. The analysis here also serves as a helpful baseline relative to changes in the policy environment, for example, increased numbers of reimbursement agreements via the VA-IHS memorandum of understanding, or changes attendant to Veterans Community Care Program.

## Conclusions

As VA considers the viability of community partners, assessing increased partnership with IHS facilities, often the only accessible health care option for rural AIAN veterans, will be critical. Our results indicate the viability of such partnerships, with the added benefit of likely improving care for all patients that IHS serves. These analyses also reveal the challenge of coordinating care efficiently and effectively across systems; efforts to link veterans’ health records so health care providers can access medical histories and current treatment plans will most certainly improve care for veterans.

## Supporting information

S1 File Indian Health Service Improving Health Care Delivery Data project infrastructure: Sources of data, key variables, and supplemental table. (DOCX)
