# Supplementary material for: American Indian and Alaska Native veterans in the Indian Health Service: Health status, utilization, and cost
Source: PLoS One. 2022 Apr 1;17(4):e0266378. doi: 10.1371/journal.pone.0266378 (PMC8975153; doi:10.1371/journal.pone.0266378)
Supplement: S1 File — (DOCX) [file pone.0266378.s001.docx]

**Supplemental information**

**Indian Health Service Improving Health Care Delivery Data project infrastructure: Sources of data, key variables, and supplemental table**

This appendix includes supplemental information on the data and methods utilized in this study including (1) an overview of the data infrastructure for the analyses; (2) a description of the key variables of the data infrastructure; and (3) a supplemental table of health coverage and hospital utilization. The text includes wording provided in the Methods section of the manuscript to provide context for the additional information provided only in this document.

1. **Data Source**

The Indian Health Service (IHS) *Improving Health Care Delivery Data Project* (IHS Data Project) includes information on a purposeful sample of American Indian and Alaska Native (AIAN) peoples who accessed IHS funded services and lived in one of 15 IHS Service Units (hereafter referred to a project sites). As a result of numerous treaty agreements, based in part on the exchange for tribal lands and natural resources, the U.S. government has a trust responsibility to provide health services for AIANs. To meet this responsibility, the U.S. government established the IHS to provide health services at no cost to members of federally recognized tribes.1 Tribal members are eligible to use IHS funded services (e.g., inpatient, outpatient, and home services) throughout their lifetime.

The IHS service delivery system includes IHS operated hospitals and clinics, tribally operated hospital and clinics, and urban Indian clinics. Of the 15 IHS project sites included in the IHS Data Project, 9 are IHS direct service programs and 6 are tribally operated Health Programs. The IHS Data Project includes little data for urban Indian clinics. The IHS Data Project includes little data for urban Indian clinics; we refer to IHS and tribal (I/T) services and other (non-I/T) services below. I/T hospitals and clinics obtain supplemental funding from IHS that includes reimbursement from Medicare, Medicaid, Veterans Health Administration, and private insurers for I/T provided services; funding from tribes; and grants.

The IHS electronic data sources included in the IHS Data Project’s data infrastructure are the: 1) National Data Warehouse (NDW) for I/T utilization data, 2) Purchased/Referred Care (PRC) for PRC utilization and payments for services obtained at non-I/T providers, and 3) Centers for Medicare & Medicaid Services Cost Reports for site-specific data on the costs of providing I/T services. Figure 1 provides information on these data sources and the key variables, described below, are created from the data.

1. **Description of Key Variables in the Data Infrastructure**

Each patient has a unique computer-generated number that we used to link their NDW registration data, NDW I/T service utilization data, and PRC utilization and payment data. Below we provide additional information on key measures and a data flow diagram (S1 Fig) that describes the data linkages.

Demographic and health coverage. NDW registration data provided information on age, gender, project site, health coverage (e.g., Medicare, Medicaid, private insurance), and veteran status. If a patient did not have any type of health coverage during FY2013, we created a measure that indicated the patient had ‘no coverage other than access to IHS/tribal services.”

Service Utilization. Information on I/T inpatient and outpatient service utilization was obtained from the NDW data; PC data provided information on non-I/T service use.

Treatment costs. FY2013 IHS treatment cost estimates were derived from FY2013 Cost Report data, FY2013 NDW I/T utilization data, and FY2013 PRC payment data for non-I/T services paid by IHS and Tribal health programs. Below we describe our method for estimating a patient’s FY2013 costs for hospital inpatient and emergency department (ED) services, outpatient utilization excluding ED services, and prescribed medications and calculating their estimated FY2013 total treatment costs.

Each fiscal year, IHS financial consultants compile data on the costs of operating the I/T hospitals and clinics in project sites in site-specific Cost Reports, using government accounting practices. The Cost Report I/T data are used to create Medicare and Medicaid reimbursement rates for I/T-provided services (e.g., one inpatient day, one outpatient visit). The Cost Reports include costs for personnel salaries and benefits, facilities, equipment, operational costs (e.g., heating, electricity), supplies, and medications.

To estimate the average cost for each type of I/T service at a project site, we estimated and summed 1) the average facility cost, 2) average personnel costs, 3) average ancillary service costs (e.g., laboratory, radiology), and 4) other service-related costs (e.g., supplies, travel time for home visits), using NDW utilization data and site-specific Cost Report data, and supplemental information obtained from existing project site reports, ad hoc project site reports, and expert opinion. For each site, we estimated site-specific costs for each of the following I/T provided services: one inpatient day; one emergency, urgent care, primary care, specialty care including ambulatory surgery, optometry/ ophthalmology, foot care, behavioral health, dental, health education, case management, clinical pharmacy, physical therapy and other rehabilitative services, nursing, and other type of office visit; home visit; and one dispensed medication.

For each adult in the study population, we estimated their costs for I/T-provided services based on their utilization of I/T services (e.g., 1 ED visit, 2 primary care visits, 5 medications) during FY2013 and the estimated average cost of providing each of those services in the site where they lived during the same year (e.g., the cost of an ED visit, a primary care visit, a medication). For each adult, we used the PRC-paid amounts for the non-I/T services they utilized (e.g., hospital inpatient stay, ED visit, outpatient visit) as the estimated IHS costs for non-I/T services. In the manuscript, we refer to IHS total treatment costs for each adult as the sum of their estimated costs for I/T- and non-I/T-provided services.

Costs for services from non-I/T providers that were not paid by the PRC program (e.g., specialty inpatient and outpatient services, renal dialysis) were not available and, thus, not included. These analyses also exclude tribal costs associated with many home visits and nearly all nursing home services.


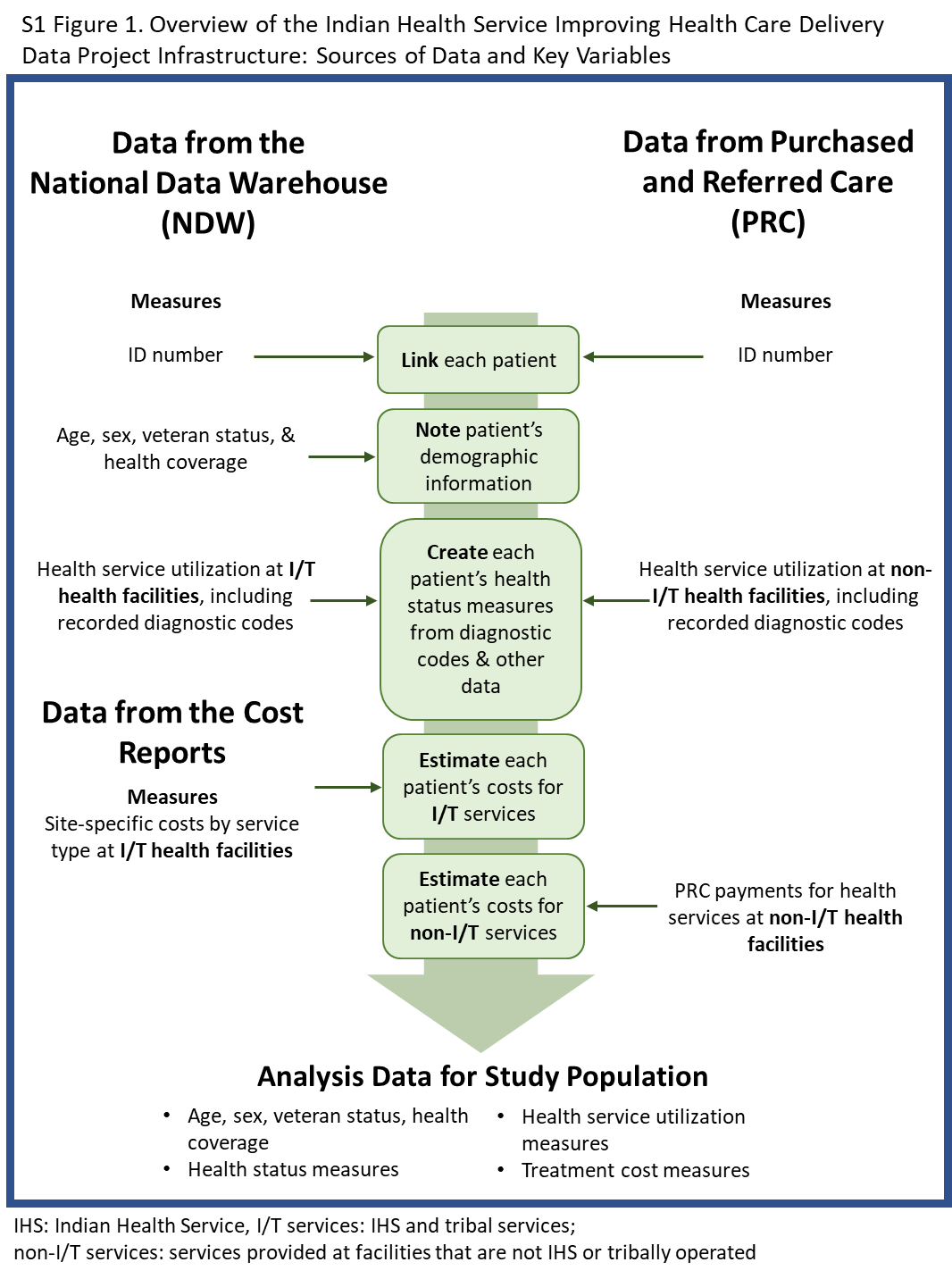


1. **Results**

S1 Table includes information on health coverage of the study population by veteran status. In this table, we also provide additional information on their use of hospital inpatient services.

| **S1 Table. Health coverage and hospital utilization among American Indian/Alaska Native veterans and non-veterans. Fiscal year 2013. All project sites.** | | | | |
| --- | --- | --- | --- | --- |
| **Health coverage: Adults with no coverage other than IHS access^a^** | | | | |
| **Age group** | **Veterans** | | **Non-Veterans** | |
|  | **Number** | **Percent** | **Number** | **Percent** |
| 18-34 | 637 | 65.9% | 661 | 70.5% |
| 35-44 | 721 | 64.8% | 801 | 71.3% |
| 45-54 | 1,125 | 66.5% | 1,195 | 70.3% |
| 55-64 | 1,385 | 60.0% | 1,530 | 65.7% |
| 65+ | 206 | 5.1% | 288 | 7.2% |
| All ages | 4,074 | 40.4% | 4,475 | 44.3% |
| **Hospital inpatient utilization** | | | | |
|  | **Veterans** | | **Non-Veterans** | |
|  | **Number** | **Percent** | **Number** | **Percent** |
| **Hospital admissions** |  |  |  |  |
| I/T admissions | 548 | 46.2% | 626 | 49.2% |
| Non-I/T admissions | 639 | 53.8% | 646 | 50.8% |
| Total admissions | 1,187 | 100.0% | 1,272 | 100.0% |
| **Hospital days** |  |  |  |  |
| I/T days | 2,947 | 56.2% | 3,208 | 54.5% |
| Non-I/T days | 2,293 | 43.8% | 2,675 | 45.5% |
| Total days | 5,240 | 100.0% | 5,883 | 100.0% |
| ^a^Data are reported for adults who used IHS services in fiscal year 2013. The total number of veterans of all ages was 10,094. The number of non-veterans of all ages was 10,106. | | | | |
